# A multicenter retrospective cohort study on the efficacy and safety of mycophenolate mofetil plus hydroxychloroquine therapy in IgA nephropathy

**DOI:** 10.3389/fimmu.2026.1783946

**Published:** 2026-03-18

**Authors:** Yang Yang, Jing Ning, Fang Zeng, Wenjun Yan, Kaiping Luo, Baoqin Zhou, Lijuan Wang, Shizhang Xu, Shufang Fu, Daijin Ren, Gaosi Xu

**Affiliations:** 1 Department of Nephrology, The Second Affiliated Hospital, Jiangxi Medical College, Nanchang University, Nanchang, China; 2 Department of Nephrology, The Affiliated Ganzhou Hospital, Jiangxi Medical College, Nanchang University, Ganzhou, China; 3 Department of Nephrology, The First Affiliated Hospital of Gannan Medical University, Ganzhou, China; 4 Department of Nephrology, Xinyu People’s Hospital, Xinyu, China; 5 Department of Nephrology, Shangrao People’s Hospital, Shangrao, China; 6 Department of Nephrology, Yichun People’s Hospital, Yichun, China; 7 Department of Ultrasound, The First Affiliated Hospital, Jiangxi Medical College, Nanchang University, Nanchang, China; 8 Department of Health Management Center, Jiangxi Provincial People’s Hospital, The First Affiliated Hospital of Nanchang Medical College, Nanchang, China

**Keywords:** hydroxychloroquine, immunoglobulin a nephropathy, mycophenolate mofetil, proteinuria, remission

## Abstract

**Background:**

The mechanisms of action of mycophenolate mofetil (MMF) and hydroxychloroquine (HCQ) differ in the treatment of IgA nephropathy (IgAN), and the two may have a synergistic effect in delaying disease progression.

**Methods:**

This multicenter retrospective cohort study included patients aged 18–60 years with biopsy-confirmed primary IgAN, with 83 patients receiving MMF plus HCQ (combined group) and 94 receiving MMF alone (MMF group). All patients had an estimated glomerular filtration rate > 45 ml/min/1.73 m^2^ and urine protein (UP) >0.75 g/d after receiving renin-angiotensin-aldosterone system inhibitors for more than 4 weeks. Propensity score matching was performed with a matching ratio of 1:1. The primary outcomes were complete response (CR) and overall remission (OR) rates at 12 months, which were analyzed using the chi-square test. CR was defined as a 24-hour UP ≤0.3 g/d and stable renal function. The OR comprised CR and partial remission. Partial remission was defined as a >50.0% reduction from baseline in UP, with a final level of <1 g/d.

**Results:**

After matching, the CR rates at 12 months were 63.8% (37/58) and 37.9% (22/58) in the combination and MMF groups, respectively (odds ratio: 0.59, 95% CI 0.40–0.87, *P* = 0.005). OR was achieved by 53 (91.4%) and 43 (74.1%) participants in the combined and MMF groups, respectively (odds ratio: 0.45, 95% CI 0.21–0.99, *P* = 0.014). Kaplan–Meier analysis also showed that the probability of achieving CR and OR was significantly higher in the combination group (log-rank *P* = 0.008 and 0.001, respectively). Subgroup analyses showed that patients with UP >2 g/d and eGFR <60 mL/min/1.73 m^2^ were more likely to achieve OR. The incidence of adverse events was comparable between the two groups.

**Conclusion:**

Combination therapy with MMF and HCQ was associated with higher remission rates and greater reduction in UP at 12 months, particularly among those with baseline UP >2 g/d or eGFR between 45 and 60 mL/min/1.73 m², supporting its potential short-term renal benefit.

## 1 Introduction

Immunoglobulin A nephropathy (IgAN) is a common form of primary glomerulonephritis worldwide. IgAN occurs more in Asian populations than in Caucasian populations (1). In China, IgAN is the most prevalent form of primary glomerulonephritis, accounting for 44.4% of cases (2).

IgAN development is associated with the abnormal production of galactose-deficient IgA1 (Gd-IgA1), which requires antigen-presenting cells to provide key activating signals. Hydroxychloroquine (HCQ) exerts anti-inflammatory and immunomodulatory effects by blocking toll-like receptors (TLRs) on antigen-presenting cells and has been widely used in the treatment of autoimmune diseases (3). Mycophenolate mofetil (MMF) is a purine antagonist that exerts a degree of inhibitory effect on the proliferation of B and T lymphocytes (4). Importantly, the 2025 Kidney Disease: Improving Global Outcomes (KDIGO) guidelines indicate that both HCQ and MMF may exert potential therapeutic effects in Chinese IgAN patients (5), as both significantly reduce proteinuria in Chinese patients with IgAN (6–9).

However, it remains unclear whether combination therapy with HCQ and MMF offers greater therapeutic benefits than monotherapy in patients with IgAN. Considering the potential synergistic effects arising from the different therapeutic mechanisms of the two agents, we hypothesized that combination therapy may yield more pronounced reductions in proteinuria. Therefore, this study aimed to evaluate the efficacy and safety of the combination of MMF and HCQ in the treatment of IgAN.

## 2 Methods

### 2.1 Study population

We conducted a retrospective, multicenter cohort study at five research centers in Jiangxi Province, China from March 2023 to July 2024. The inclusion criteria were as follows (1): age between 18 and 60 years (2); biopsy-proven primary IgAN (3); patients treated with either a combination of MMF and HCQ or MMF alone (4); 24-hour urine protein (UP) ≥0.75 g/day after receiving renin-angiotensin-aldosterone system inhibitors for more than four weeks (5); estimated glomerular filtration rate (eGFR) ≥45 mL/min/1.73 m^2^, calculated using the Chronic Kidney Disease Epidemiology Collaboration creatinine equation (10). Patients with the following conditions were excluded: lack of relevant follow-up data, any secondary form of IgAN or IgA vasculitis or any non-IgAN glomerulonephritis, major hepatic, cerebrovascular, or cardiovascular comorbidities, prior kidney transplantation, or any prior immunosuppressive therapy. The present study was approved by the Ethics Committee of the Second Affiliated Hospital of Nanchang University, and all patients provided written informed consent (IT-O-2025-184).

### 2.2 Intervention and follow-up

All eligible patients were assigned to receive either the combined group (MMF combined with HCQ) or the MMF group (MMF monotherapy). Patients treated with MMF received an oral dose of 1.25 g–1.5 g daily for 6 months, followed by tapering to a maintenance dose of 0.75 g–1.0 g daily for 6 months. HCQ was administered orally for 12 months, with the dosage adjusted based on renal function: 0.2 g twice daily for patients with eGFR >60 mL/min/1.73 m^2^ and 0.1 g three times daily for those with an eGFR between 45 mL/min/1.73 m^2^ and 59 mL/min/1.73 m^2^. Data were collected from the participants at baseline and 1, 3, 6, 9, and 12 months after treatment.

### 2.3 Study outcome

The primary endpoints were complete response (CR) and overall remission (OR) at 12 months post-treatment. CR was defined as a 24-hour UP ≤0.3 g/d and stable renal function (a decrease in eGFR ≤30.0%). The OR comprised CR and partial remission. Partial remission was defined as a >50.0% reduction from baseline in UP, with a final level of <1 g/d. The secondary endpoints included changes in the 24-hour UP and eGFR, as well as adverse events.

### 2.4 24-hour UP collection

All participants received written and verbal instructions for collecting a 24-hour UP sample. They were instructed to discard the first-morning urine at the start of the day and then collect all subsequent UP in a container containing a preservative over the next 24-hour with the lid tightly closed. Participants were required to accurately record the start and end times of the collection and note the total volume of UP collected. After thoroughly mixing the UP samples, 10 mL was extracted and sent to the hospital for testing.

### 2.5 Statistical analysis

Continuous variables that followed a normal distribution are presented as mean ± standard deviation (SD) and were compared between groups using Student’s t-test, while non-normally distributed data are expressed as median (interquartile range IQR) and were compared using the Mann–Whitney U test. Categorical data were presented as frequencies and percentages and analyzed using the chi-squared test or Fisher’s exact test. We conducted propensity score matching (PSM) with a 1:1 ratio and a caliper of 0.2, using age, sex, baseline UP, eGFR, and serum albumin levels. The primary outcomes (CR and OR rates at 12 months) were analyzed using the chi-square test. The probability of remission was evaluated using Kaplan–Meier survival curves, and group comparisons were performed using the log-rank test. Variables identified as significant in the univariate Cox regression analysis were entered into a multivariate model to determine the adjusted hazard ratio (HR) for the 12-month remission rate. Subgroup analyses were performed using stratified Cox proportional hazards models according to baseline features, with the results displayed in a forest plot. Interactions among the subgroups were assessed using the likelihood ratio test. Statistical significance was set at *P <*0.05. Statistical analyses were performed using R (version 4.4.3) and GraphPad (version 9.0).

## 3 Results

### 3.1 Baseline characteristic

**Figure 1:**
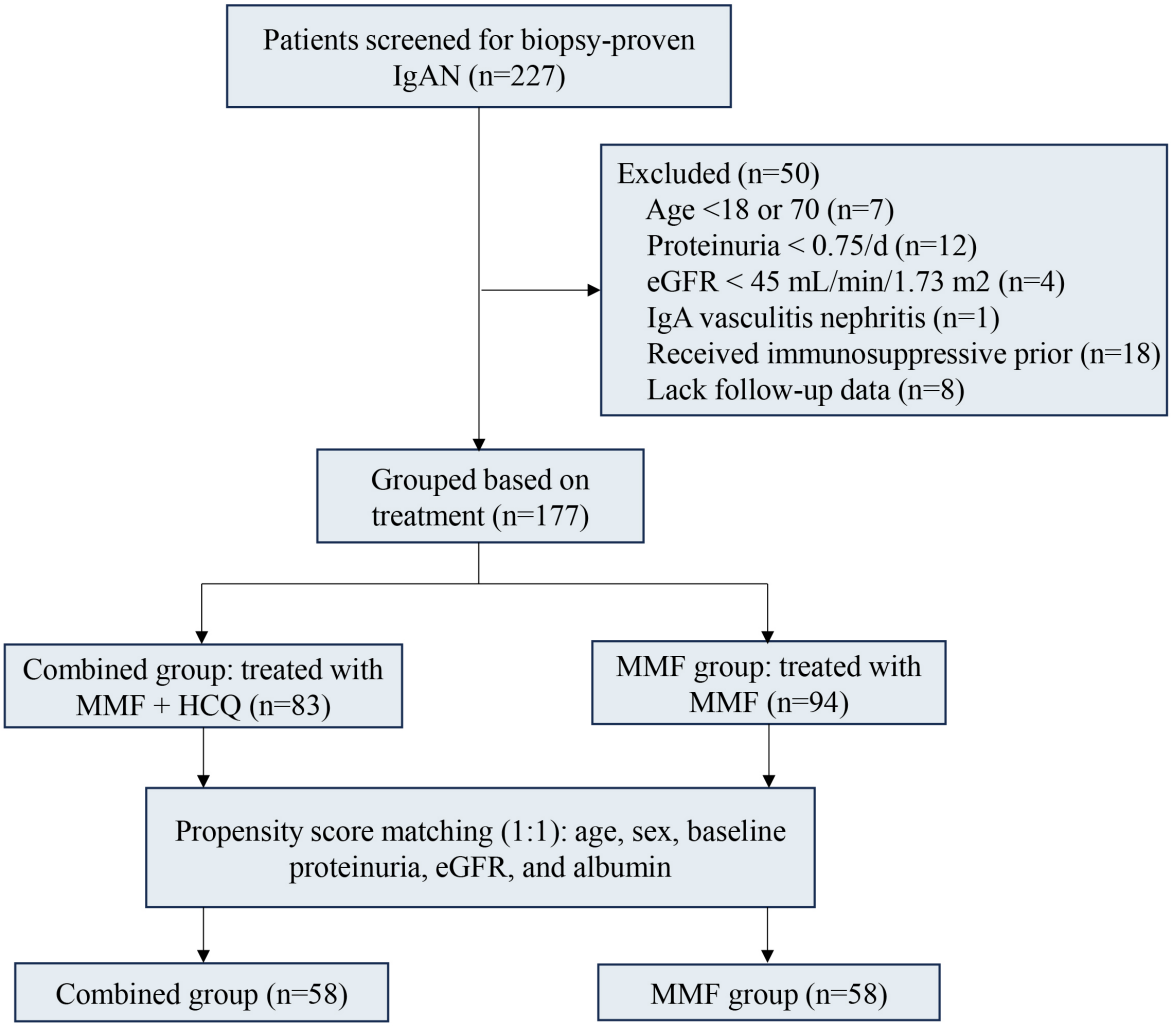
Study flowchart.

According to the inclusion and exclusion criteria, 177 eligible patients were selected from an initial cohort of 227 patients with IgAN. Among them, 83 patients received treatment with MMF combined with HCQ, whereas 94 received MMF monotherapy (**Figure 1**). Although the two patient groups were balanced at baseline, to further ensure comparability between them, we conducted PSM at a 1:1 ratio, focusing on matching age, sex, baseline UP, serum albumin, and eGFR levels, resulting in the establishment of 58 patients in the combined group and 58 in the MMF group. A comparison of the baseline characteristics between the combined and MMF groups revealed no significant differences in the median (IQR) eGFR (69.65 [55.95, 85.02] vs. 70.05 [56.24, 92.81] mL/min/1.73 m^2^, *P* = 0.897), 24-hour UP (1.76 [1.38, 3.06] vs. 1.88 [1.51, 4.30] g/d, *P* = 0.253), MEST-C scores, or other clinical indicators (**Table 1**).

**Table 1:** Baseline characteristics of patients.

Characteristic	The full cohort (N = 177)			The matched cohort (N = 116)		
	Combined group (N = 83)	MMF group (N = 94)	*P*	Combined group (N = 58)	MMF group (N = 58)	*P*
Female (n, %)	41 (49.4)	53 (56.4)	0.436	28 (48.3)	32 (55.8)	0.577
Age (years)	38.00 [31.00, 50.00]	40.00 [33.25, 48.00]	0.636	42.05 (11.91)	39.22 (10.44)	0.177
Hypertension (n, %)	21 (25.3)	23 (24.5)	>0.999	13 (22.4)	16 (27.6)	0.668
Diabetes (n, %)	4 (4.8)	4 (4.3)	>0.999	3 (5.2)	3 (5.2)	>0.999
BMI (kg/m^2^)	23.5 (3.3)	24.2 (3.7)	0.199	23.5 (3.2)	24.1 (3.6)	0.338
SBP (mmHg)	130.00 [118.00, 141.00]	129.00 [120.00, 141.50]	0.686	130.00 [118.25, 140.00]	130.50 [120.00, 140.00]	0.791
DBP (mmHg)	85.00 [76.00, 96.00]	84.50 [77.00, 93.50]	0.754	86.00 [75.25, 93.75]	85.00 [77.25, 91.75]	0.667
RBC (×10^12^/L)	4.69 (0.70)	4.53 (0.70)	0.139	4.65 (0.68)	4.61 (0.71)	0.738
TP (g/L)	68.54 [63.92, 73.12]	69.34 [64.10, 74.42]	0.438	68.57 [63.76, 73.16]	68.80 [63.02, 73.07]	0.976
ALT (g/L)	15.08 [11.10, 22.91]	16.45 [11.87, 27.11]	0.303	15.39 [11.05, 22.61]	16.73 [11.33, 24.87]	0.412
AST (g/L)	19.70 [15.95, 25.66]	20.20 [17.05, 25.23]	0.751	18.93 [15.90, 24.50]	18.27 [16.30, 24.12]	0.897
BUN (mmol/L)	5.20 [4.38, 6.72]	5.60 [4.03, 7.45]	0.691	5.64 [4.50, 6.91]	5.05 [3.91, 7.22]	0.232
UA (umol/L)	383.79 (94.79)	390.92 (109.68)	0.646	381.68 (95.02)	379.02 (94.71)	0.880
TC (mmol/L)	4.98 [4.44, 5.59]	5.20 [4.59, 6.02]	0.351	5.05 [4.57, 5.48]	5.08 [4.21, 5.99]	0.910
TG (mmol/L)	1.60 [1.10, 2.37]	1.67 [1.07, 2.49]	0.581	1.69 [1.15, 2.55]	1.75 [1.15, 2.53]	0.785
HDL (mmol/L)	1.32 [1.04, 1.58]	1.31 [1.08, 1.55]	0.805	1.26 [1.10, 1.58]	1.25 [1.08, 1.53]	0.789
LDL (mmol/L)	3.08 [2.59, 3.46]	3.24 [2.54, 3.89]	0.148	3.08 [2.77, 3.47]	3.12 [2.45, 3.81]	0.757
Glu (mmol/L)	5.02 [4.50, 5.47]	5.00 [4.70, 5.53]	0.782	5.06 [4.51, 5.51]	5.04 [4.76, 5.48]	0.956
Proteinuria (g/d)	1.75 [1.40, 3.26]	1.89 [1.48, 3.71]	0.445	1.76 [1.38, 3.06]	1.88 [1.51, 4.30]	0.253
Alb (g/L)	39.40 [35.99, 41.15]	39.31 [35.35, 41.70]	0.570	39.61 [36.72, 41.34]	39.29 [35.12, 41.18]	0.361
eGFR (mL/min/1.73 m^2^)	71.18 [58.36, 87.25]	70.28 [56.24, 91.13]	0.848	69.65 [55.95, 85.02]	70.05 [56.24, 92.81]	0.897
M1 (n, %)	80 (96.4)	87 (92.6)	0.438	55 (94.8)	54 (93.1)	>0.999
E1 (n, %)	31 (37.4)	31 (33.0)	0.652	20 (34.5)	22 (37.9)	0.847
S1 (n, %)	54 (65.0)	63 (67.0)	0.908	37 (63.8)	36 (62.1)	>0.999
T (n, %)			0.578			0.553
0	55 (66.3)	67 (71.3)		21 (36.2)	17 (29.3)	
1/2	28 (33.7)	27 (28.7)		37 (63.8)	41 (70.7)	
C (n, %)			0.303			>0.999
0	43 (51.8)	57 (60.6)		25 (43.1)	24 (41.4)	
1/2	40 (48.2)	37 (39.4)		33 (56.9)	34 (58.6)	

Alb, serum albumin; ALT, alanine aminotransferase; AST, aspartate aminotransferase; BMI, body mass index; BUN, blood urea nitrogen; C, crescent; DBP, diastolic blood pressure; E, endocapillary hypercellularity; eGFR, estimated glomerular filtration rate; Glu, serum glucose; HDL, high-density lipoprotein; LDL, low-density lipoprotein; M, mesangial hypercellularity; S, segmental glomerulosclerosis; SBP, systolic blood pressure; T, interstitial fibrosis and tubular atrophy; TC, serum cholesterol; TG, serum triglyceride; TP, serum total protein; RBC, red blood cell; UA, serum uric acid.

### 3.2 Primary outcomes

Following PSM, 37 (63.8%) and 22 patients (37.9%) patients in the combined and MMF groups, respectively, achieved CR (odds ratio: 0.60, 95% CI 0.40–0.87, *P* = 0.005) at 12 months. Similarly, 53 (91.4%) and 43 (74.1%) participants achieved OR in the combined and MMF groups, respectively (odds ratio: 0.45, 95% CI 0.21–0.99, *P* = 0.014). In the full cohort, the combined group also demonstrated higher rates of CR and OR (odds ratio: 0.66, 95% CI 0.48–0.92, *P* = 0.012; odds ratio: 0.57, 95% CI 0.32–1.02, *P* = 0.028; respectively).

**Figure 2:**
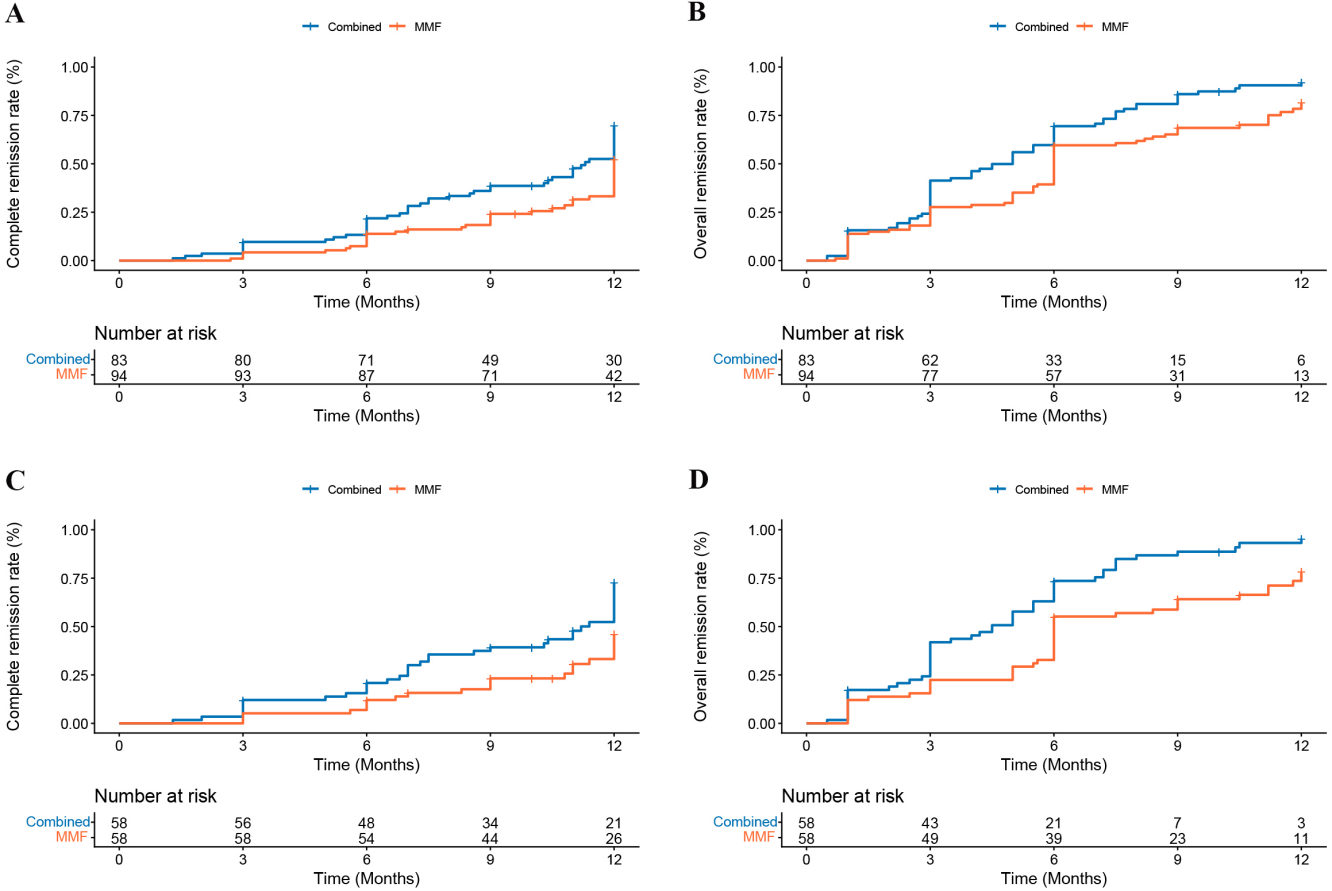
Cumulative probability of 12 months remission using Kaplan–Meier analysis. **(A)** CR (log rank *P* = 0.011) and **(B)** OR (log rank *P* = 0.004) in full cohort, **(C)** CR (log rank *P* = 0.008) and **(D)** OR (log rank *P*<0.001) in the matched cohort.

Kaplan–Meier survival analysis revealed that in both the full and matched cohorts, the combined group demonstrated significantly higher CR and OR rates than the MMF group (all log-rank *P <*0.05, **Figure 2**). After adjustment for age, UP, serum albumin, eGFR, and M and T scores, multivariate Cox regression analysis revealed that the combined group was associated with a higher likelihood of achieving 12-month CR in both the full and matched cohorts (HR: 0.57, 95% CI 0.37–0.87, *P* = 0.009; HR: 0.57, 95% CI 0.33–0.98, *P* = 0.043; respectively) (**Supplementary Table 1**). Similarly, multivariate analyses revealed that compared with combined treatment, MMF decreased the rate of OR by 38.0% and 49.0% in the full and matched cohorts, respectively (**Supplementary Table 2**).

### 3.3 Secondary outcomes

**Figure 3:**
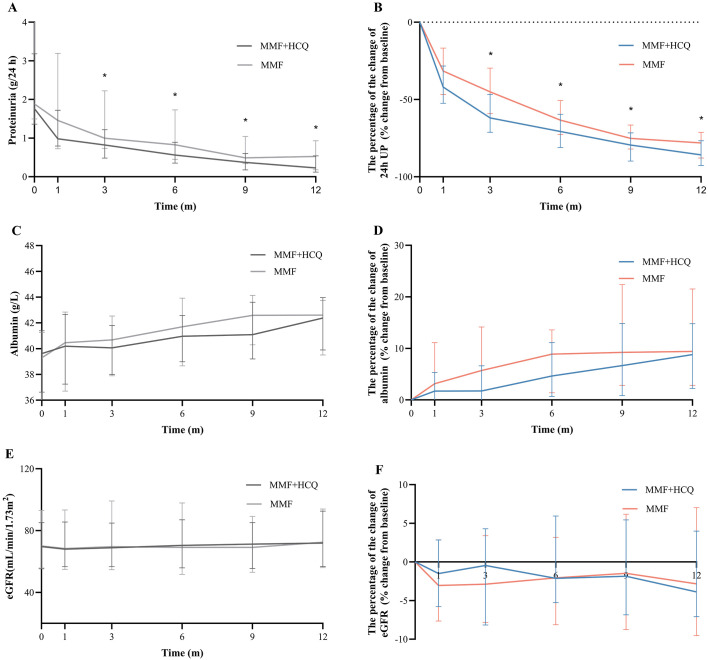
Changes in **(A, B)** proteinuria, **(C, D)** albumin, **(E, F)** eGFR in the matched cohort.

In the matched cohort, statistically significant differences in UP levels were observed between the two groups during follow-up (both *P <*0.05, **Figure 3A**). In terms of percentage change from baseline at 3 months, UP decreased by 61.9% and 45.0% in the combined and MMF groups, respectively (*P <*0.001). At the 6, 9, and 12 months, the decrease was also more pronounced in the combined group (−70.7 [IQR −81.1, −59.6] % vs. −63.4 [IQR −72.6, −50.7] %, *P* = 0.006; −79.5 [IQR −89.9, −71.6] % vs. −75.1 [IQR −82.0, −66.5] %, *P* = 0.023; −85.8 [IQR −92.7, −76.7] % vs. −78.1 [IQR −87.9, −71.3] %, *P* = 0.040, respectively) (**Figure 3B**). A similar percentage reduction in UP was observed in the full cohort (**Supplementary Figures 1A, B**). No statistical difference was observed between the two groups in the changes in eGFR and serum albumin levels at each follow-up visit compared with the baseline (**Supplementary Figures 1C–F**; **Figures 3C–F**).

### 3.4 Subgroup analysis for 12-month CR

**Figure 4:**
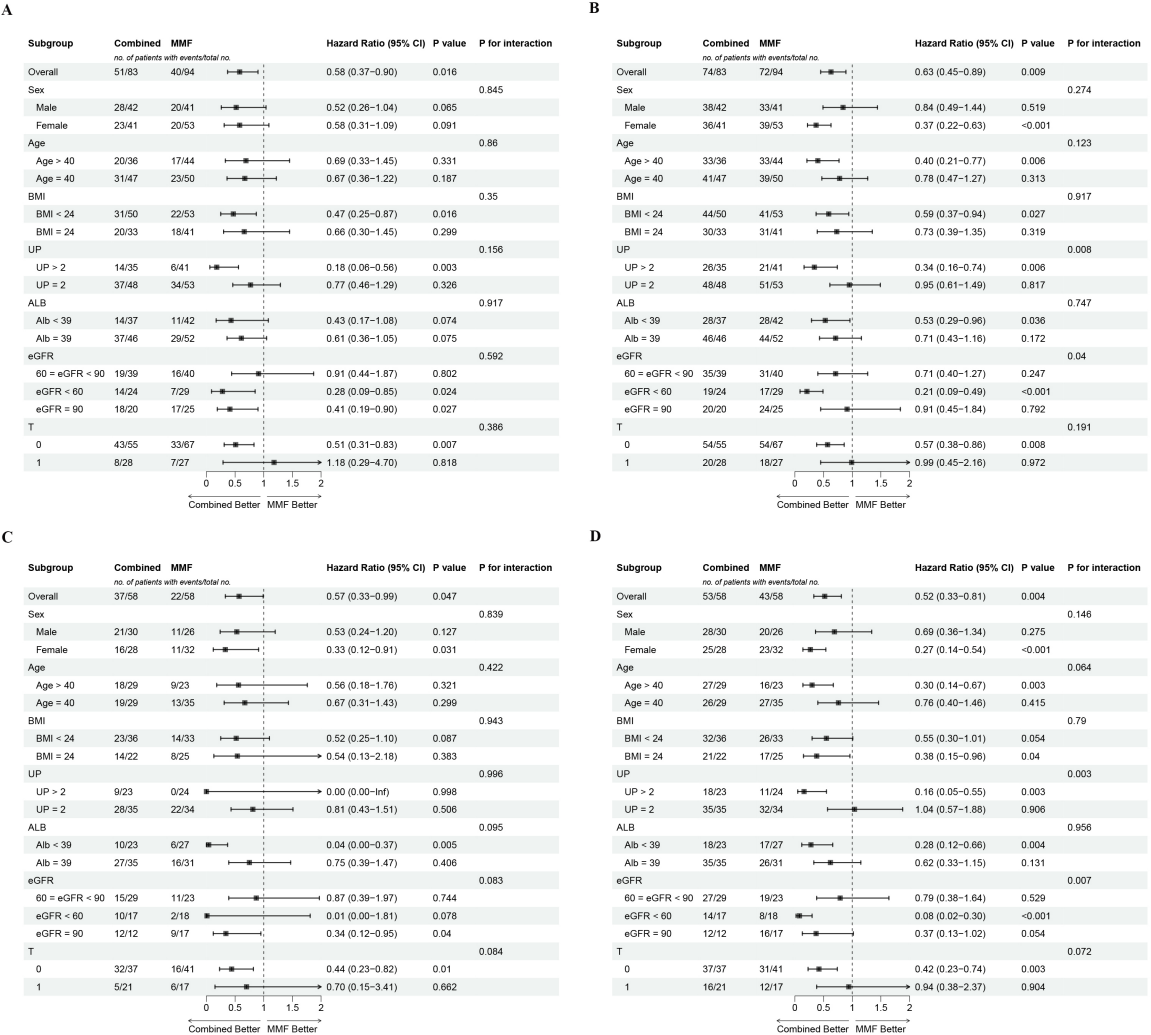
Subgroup analyses of the association between treatment regimen and 12 months complete remission and overall remission. **(A)** CR and **(B)** OR in full cohort, **(C)** CR and **(D)** OR in matched cohort. Adjusted for age, sex, BMI, proteinuria, eGFR, albumin, hypertension, diabetes, and M, E, S, T, and C scores.

After adjusting for age, sex, BMI, UP, eGFR, serum albumin, and MEST-C scores, subgroup analyses consistently demonstrated a benefit in the combined group, with an HR <1 across all prespecified subgroups. Across all subgroups examined (age, sex, renal function, serum albumin, or T), no significant interaction with CR was detected (all *P*-interaction >0.05), indicating that the treatment effect of combination therapy was consistent (**Figure 4C**). Interestingly, interaction analysis revealed that the effect of combination therapy on OR was significantly modified by baseline UP (*P*-interaction = 0.003) and eGFR (*P*-interaction = 0.007). Specifically, patients with UP >2 g/d (*P* = 0.003) or eGFR between 45 mL/min/1.73 m² and 60 mL/min/1.73 m² (*P <*0.001) were more likely to achieve an OR with this therapy (**Figure 4D**). The same results were observed in the cohort before PSM (**Figures 4A, B**).

### 3.5 Safety and adverse events

The incidence of adverse events was comparable between the two groups. No deaths or serious adverse events occurred in either group. Infections and gastrointestinal symptoms were the most common adverse reactions in both groups. Two patients in the combined group presented with skin pigmentation (**Table 2**).

**Table 2:** Adverse events.

Events	Combined group (N = 83)	MMF group (N = 94)	*P*
Infections	17 (20.5)	19 (20.2)	0.965
Pneumonia	5 (6.0)	7 (7.4)	0.707
Upper respiratory tract infection	7 (8.4)	7 (7.4)	0.808
Urinary tract infection	2 (2.4)	3 (3.2)	>0.999
Other infections	3 (3.6)	2 (2. 1)	0.666
Hepatic dysfunction	4 (4.8)	6 (6.4)	0.752
Gastrointestinal symptoms	11 (13.3)	15 (16.0)	0.612
Rash	4 (4.8)	5 (5.3)	>0.999
Skin pigmentation	2 (2.4)	0 (0)	0.218

## 4 Discussion

This multicenter, retrospective study aimed to evaluate the efficacy and safety of MMF combined with HCQ in Chinese patients with IgAN for the first time. The results demonstrated that the results demonstrated that combination therapy significantly improved clinical outcomes. Compared with MMF, the combination of MMF and HCQ achieved higher CR (63.8% vs. 37.9%, odds ratio: 0.60, 95% CI 0.40–0.87, *P* = 0.005) and OR (91.4% vs. 74.1%, odds ratio: 0.45, 95% CI 0.21–0.99, *P* = 0.014) rates at 12 months, respectively. The treatment effect was particularly pronounced in patients with baseline UP >2 g/d and those with eGFR 45 mL/min/1.73 m² –60 mL/min/1.73 m², suggesting a clinically meaningful benefit in these subgroups. Moreover, combination therapy did not increase the incidence of adverse events.

Currently, MMF and HCQ are potentially useful strategies for treating IgAN in Chinese patients. The innate immune response, particularly the activation, plays a pivotal role in IgAN pathogenesis. TLRs activation initiates downstream signaling pathways, stimulating the release of multiple cytokines and the production of Gd-IgA1, thereby driving the progression of IgAN (11–13). As a TLRs inhibitor, HCQ suppresses the proteolytic maturation of TLR9/7 and prevents ligand binding. This reduces the production of Gd-IgA1 and delays the progression of IgAN (14, 15). Liu et al. indicated that, compared with the placebo group, HCQ treatment led to a clinically meaningful reduction in UP in IgAN patients after 6 months (0.9 [IQR, 0.6, 1.0] g/d vs. 1.9 [IQR, 0.9, 2.6] g/d; *P* = 0.002, respectively) (16). A recent study also demonstrated the efficacy of HCQ in reducing UP in patients with IgAN, with a reduction of 70.4% (57.5%–79.3%) from baseline (17). MMF inhibits the proliferation of activated T/B lymphocytes and mesangial cells by suppressing the rate-limiting enzyme of *de novo* purine synthesis (18, 19). It also exerts a protective effect on podocytes, and patients receiving MMF demonstrate a marked renal survival advantage (6, 8). Therefore, this combination therapy may simultaneously target the production of Gd-IgA1 and the downstream cellular immune response.

Although the precise molecular mechanisms underlying this synergistic effect were not investigated in the present study, our clinical data indicated that combining HCQ with MMF may offer superior antiproteinuric benefits, with a decrease from 2.58 g/d at baseline to 0.38 g/d at 12 months. The observed rapid and sustained reduction in proteinuria in the combination group implies that HCQ may complement the immunosuppressive effects of MMF, potentially addressing residual disease activity in patients who do not fully respond to MMF alone, especially in patients with UP >2 g/d or eGFR between 45 mL/min/1.73 m^2^ and 60 mL/min/1.73 m^2^. These findings highlight the potential value of this combination therapy as an optimized treatment strategy for Chinese IgAN patients. However, despite the significant improvement in the 12-month remission rates with combination therapy, no significant difference was observed in eGFR decline, highlighting the need for longer-term observation. Whether this early proteinuria response ultimately translates into improved long-term renal survival remains to be validated in future studies with extended follow-up periods.

The safety profiles of both groups were favorable and similar to those reported in earlier studies (6, 16). However, several limitations of this study should be acknowledged. First, its small sample size and short follow-up period preclude clarification of the long-term clinical outcomes of these patients. Second, the retrospective nature of the study may have introduced potential bias. Third, given the therapeutic restrictions imposed by eGFR and the very limited number of patients with an eGFR between 30 and 45 receiving either MMF or HCQ treatment, this study exclusively enrolled Chinese patients with eGFR >45 mL/min/1.73 m^2^. Therefore, it is not possible to determine the applicability of this conclusion to other racial groups or individuals with an eGFR below 45. Future studies, including multicenter randomized controlled trials across different cohorts with extended follow-up periods, are needed to validate our findings and better understand the long-term impact of MMF combined with HCQ in IgAN.

In conclusion, the combination therapy regimen of MMF and HCQ was associated with higher remission rates and greater reduction in UP at 12 months, suggesting that it may offer short-term renal benefits. For patients with UP >2 g/day or an eGFR between 45 mL/min/1.73 m²and 60 mL/min/1.73 m², combination therapy was more likely to achieve OR. Further randomized trials are required to confirm these observations.

## Data Availability

The original contributions presented in the study are included in the article/**Supplementary Material**. Further inquiries can be directed to the corresponding author.

## Supplementary material

Supplementary Figure 1 Changes in **(A, B)** proteinuria, **(C, D)** albumin, **(D, E)** eGFR in the full cohort. Represented indirectly by the median and quartiles.

Supplementary Table 1 Univariate and multivariate cox regression analyses of the complete remission in the cohort study and after propensity score matching.

Supplementary Table 2 Univariate and multivariate cox regression analyses of the overall remission in the cohort study and after propensity score matching.

## References

[1] Schena FP Nistor I . Epidemiology of igA nephropathy: A global perspective. Semin Nephrol. (2018) 38:435–42. doi: 10.1016/j.semnephrol.2018.05.013, PMID: 30177015

[2] Wang Y Zhang L Yuan L Xie Q Liu S Hao CM . Changes in the spectrum of biopsy-proven renal diseases over 11 years: a single-center study in China. Ren Fail. (2024) 46(2):2381614. doi: 10.1080/0886022X.2024.2381614, PMID: 39039852 PMC11268216

[3] Li HZ Xu XH Lin N Lu HD . Metabolic and cardiovascular benefits of hydroxychloroquine in patients with rheumatoid arthritis: a systematic review and meta-analysis. Ann Rheum Dis. (2019) 78:e21. doi: 10.1136/annrheumdis-2018-213157, PMID: 29453218

[4] Sepe V Libetta C Giuliano MG Adamo G Dal Canton A . Mycophenolate mofetil in primary glomerulopathies. Kidney Int. (2008) 73:154. doi: 10.1038/sj.ki.5002653, PMID: 17989649

[5] Rovin BH Barratt J Cook HT Noronha IL Reich HN Suzuki Y . KDIGO 2025 clinical practice guideline for the management of immunoglobulin A nephropathy (IgAN) and immunoglobulin A vasculitis (IgAV). Kidney Int. (2025) 108:S1–S71. doi: 10.1016/j.kint.2025.04.004, PMID: 40975564

[6] Hou FF Xie D Wang J Xu X Yang X Ai J . Effectiveness of mycophenolate mofetil among patients with progressive IgA nephropathy. JAMA Netw Open. (2023) 6(2):e225054. doi: 10.1001/jamanetworkopen.2022.54054, PMID: 36745456 PMC12578496

[7] Tang C Lv J-C Shi S-F Chen Y-Q Liu L-J Zhang H . Long-term safety and efficacy of hydroxychloroquine in patients with IgA nephropathy: a single-center experience. J Nephrol. (2021) 35(2):429–40. doi: 10.1007/s40620-021-00988-1, PMID: 33591553

[8] Tang SCW Tang AWC Wong SSH Leung JCK Ho YW Lai KN . Long-term study of mycophenolate mofetil treatment in IgA nephropathy. Kidney Int. (2010) 77(6):543–9. doi: 10.1038/ki.2009.499, PMID: 20032964

[9] Yang T Shi Y Wang Y Feng Y Shao Q Jiang C . Hydroxychloroquine blood concentrations and effects in Chinese patients with IgA nephropathy. J Nephrol. (2024) 37(8):2201–8. doi: 10.1007/s40620-024-02029-z, PMID: 39048780 PMC11649793

[10] Levey AS Stevens LA Schmid CH Zhang YL Castro AF Feldman HI . A new equation to estimate glomerular filtration rate. Ann Intern Med. (2009) 150(9):604–12. doi: 10.7326/0003-4819-150-9-200905050-00006, PMID: 19414839 PMC2763564

[11] Chen R Zou J Chen J Zhong X Kang R Tang D . Pattern recognition receptors: function, regulation and therapeutic potential. Signal Transduct Target Ther. (2025) 10(1):216. doi: 10.1038/s41392-025-02264-1, PMID: 40640149 PMC12246121

[12] Lee M Suzuki H Ogiwara K Aoki R Kato R Nakayama M . The nucleotide-sensing Toll-Like Receptor 9/Toll-Like Receptor 7 system is a potential therapeutic target for IgA nephropathy. Kidney Int. (2023) 104(5):943–55. doi: 10.1016/j.kint.2023.08.013, PMID: 37648155

[13] Vincent FB Saulep-Easton D Figgett WA Fairfax KA Mackay F . The BAFF/APRIL system: emerging functions beyond B cell biology and autoimmunity. Cytokine Growth Factor Rev. (2013) 24(3):203–15. doi: 10.1016/j.cytogfr.2013.04.003, PMID: 23684423 PMC7108297

[14] Yilei L Yating D Yaxuan F Chenxuan L Tingzhu C Jinpu L . Hydroxychloroquine sulfate for IgA nephropathy: mechanisms and therapeutic potential in improving proteinuria and alleviating disease progression - a literature review. BMC Nephrol. (2025) 26(1):317. doi: 10.1186/s12882-025-04262-5, PMID: 40597811 PMC12210545

[15] Fava A Petri M . Systemic lupus erythematosus: Diagnosis and clinical management. J Autoimmun. (2019) 96:1–13. doi: 10.1016/j.jaut.2018.11.001, PMID: 30448290 PMC6310637

[16] Liu L-J Yang Y-z Shi S-F Bao Y-F Yang C Zhu S-N . Effects of hydroxychloroquine on proteinuria in IgA nephropathy: A randomized controlled trial. Am J Kidney Dis. (2019) 74(1):15–22. doi: 10.1053/j.ajkd.2019.01.026, PMID: 30922594

[17] He W-j Wang J Liu N Li G-y Zhu X-w Yao L . The efficacy and safety of hydroxychloroquine versus leflunomide in patients with IgA nephropathy: a single-center experience. J Nephrol. (2024) 37(4):933–40. doi: 10.1007/s40620-023-01839-x, PMID: 38225440 PMC11239748

[18] Hackl A Ehren R Weber LT . Effect of mycophenolic acid in experimental, nontransplant glomerular diseases: new mechanisms beyond immune cells. Pediatr Nephrol. (2017) 32(8):1315–22. doi: 10.1007/s00467-016-3437-y, PMID: 27312386

[19] El Karoui K Fervenza FC De Vriese AS . Treatment of IgA nephropathy: A rapidly evolving field. J Am Soc Nephrol. (2024) 35(1):103–16. doi: 10.1681/asn.0000000000000242, PMID: 37772889 PMC10786616

