# Supplementary material for: A multicenter retrospective cohort study on the efficacy and safety of mycophenolate mofetil plus hydroxychloroquine therapy in IgA nephropathy
Source: Front Immunol. 2026 Mar 18;17:1783946. doi: 10.3389/fimmu.2026.1783946 (PMC13038614; doi:10.3389/fimmu.2026.1783946)
Supplement: Supplementary Figure 1 — Changes in (A, B) proteinuria, (C, D) albumin, (D, E) eGFR in the full cohort. Represented indirectly by the median and quartiles. [file DataSheet1.docx]

**Supplementary material**

**Supplementary Figure 1: Changes in (A, B) proteinuria, (C, D) albumin, (D, E) eGFR in the full cohort.**

**Supplementary Table 1 Univariate and multivariate cox regression analyses of the complete remission in the cohort study and after propensity score matching.**

**Supplementary Table 2. Univariate and multivariate cox regression analyses of the overall remission in the cohort study and after propensity score matching**


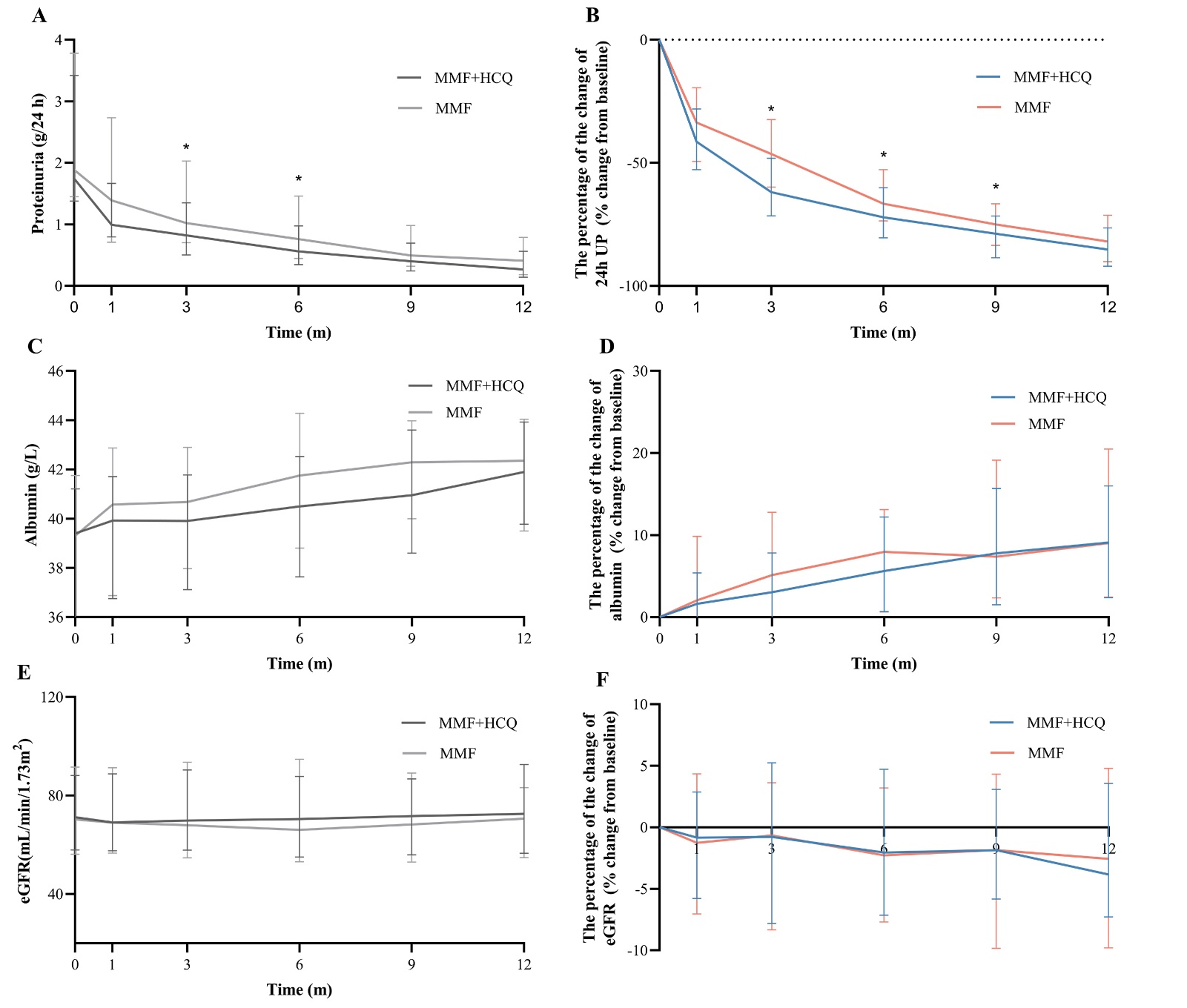


**Supplementary Figure 1: Changes in (A, B) proteinuria, (C, D) albumin, (D, E) eGFR in the full cohort.** Represented indirectly by the median and quartiles.

**Supplementary Table 1. Univariate and multivariate cox regression analyses of the complete remission in the cohort study and after propensity score matching**

| **Characteristic** | **The full cohort (n=177)** | | | | **The matched cohort (n=116)** | | | |
| --- | --- | --- | --- | --- | --- | --- | --- | --- |
|  | **Univariate analyses** | | **Multivariate analyses** | | **Univariate analyses** | | **Multivariate analyses** | |
|  | **HR (95% CI)** | ***P*** | **HR (95% CI)** | ***P*** | **HR (95% CI)** | ***P*** | **HR (95% CI)** | ***P*** |
| Treatment (MMF) | 0.59 (0.39-0.90) | 0.013 | 0.57 (0.37-0.87) | 0.009 | 0.49 (0.29-0.84) | 0.009 | 0.57 (0.33-0.98) | 0.043 |
| Age (per year) | 0.98 (0.96-1.00) | 0.019 | 0.98 (0.96-0.99) | 0.013 | 0.98 (0.96-1.01) | 0.223 | 0.98 (0.96-1.01) | 0.169 |
| Gender (male) | 0.74 (0.49-1.11) | 0.145 |  |  | 0.68 (0.41-1.14) | 0.140 |  |  |
| BMI (kg/m^2^) | 0.98 (0.92-1.04) | 0.554 |  |  | 0.96 (0.89-1.04) | 0.351 |  |  |
| Proteinuria (g/d) | 0.58 (0.47-0.72) | <0.001 | 0.67 (0.52-0.87) | 0.002 | 0.47 (0.33-0.67) | <0.001 | 0.48 (0.32-0.71) | <0.001 |
| Alb (g/L) | 1.12 (1.08-1.16) | <0.001 | 1.06 (1.01-1.12) | 0.031 | 1.14 (1.06-1.22) | <0.001 | 0.98 (0.89-1.08) | 0.715 |
| eGFR (mL/min/1.73m^2^) | 1.01 (1.00-1.02) | 0.009 | 1.00 (0.99-1.01) | 0.437 | 1.01 (1.00-1.02) | 0.043 | 0.99 (0.98-1.00) | 0.228 |
| M1 | 0.41 (0.21-0.82) | 0.012 | 0.59 (0.28-1.26) | 0.172 | 0.53 (0.23-1.24) | 0.143 | 0.66 (0.25-1.72) | 0.393 |
| E1 | 0.83 (0.53-1.29) | 0.408 |  |  | 0.85 (0.49-1.45) | 0.541 |  |  |
| S1 | 0.74 (0.49-1.13) | 0.160 |  |  | 0.71 (0.43-1.19) | 0.191 |  |  |
| T1/2 | 0.38 (0.22-0.65) | <0.001 | 0.54 (0.31-0.95) | 0.033 | 0.38 (0.20-0.74) | 0.004 | 0.57 (0.29-1.11) | 0.097 |
| C1/2 | 1.01 (0.66-1.52) | 0.977 |  |  | 0.96 (0.57-1.61) | 0.872 |  |  |
| Hypertension | 0.96 (0.59-1.57) | 0.869 |  |  | 1.04 (0.58-1.87) | 0.900 |  |  |
| Diabetes | 0.33 (0.08-1.36) | 0.125 |  |  | 0.21 (0.03-1.49) | 0.117 |  |  |

Abbreviations: Alb: serum albumin; BMI: body mass index; C: crescent; DBP: diastolic blood pressure; E: endocapillary hypercellularity; eGFR: estimated glomerular filtration rate; M: mesangial hypercellularity; S: segmental glomerulosclerosis; T: interstitial fibrosis and tubular atrophy.

**Supplementary Table 2. Univariate and multivariate cox regression analyses of the overall remission in the cohort study and after propensity score matching**

| **Characteristic** | **The full cohort (n=177)** | | | | **The matched cohort (n=116)** | | | |
| --- | --- | --- | --- | --- | --- | --- | --- | --- |
|  | **Univariate analyses** | | **Multivariate analyses** | | **Univariate analyses** | | **Multivariate analyses** | |
|  | **HR (95% CI)** | ***P*** | **HR (95% CI)** | ***P*** | **HR (95% CI)** | ***P*** | **HR (95% CI)** | ***P*** |
| Treatment (MMF) | 0.63 (0.45-0.87) | 0.006 | 0.62 (0.44-0.86) | 0.005 | 0.48 (0.32-0.73) | <0.001 | 0.51 (0.33-0.80) | 0.003 |
| Age (per year) | 0.99 (0.98-1.01) | 0.383 |  |  | 0.99 (0.98-1.01) | 0.587 |  |  |
| Gender (male) | 0.73 (0.53-1.01) | 0.056 |  |  | 0.72 (0.48-1.07) | 0.107 |  |  |
| BMI (kg/m^2^) | 0.99 (0.94-1.04) | 0.672 |  |  | 0.96 (0.91-1.02) | 0.235 |  |  |
| Proteinuria (g/d) | 0.59 (0.51-0.68) | <0.001 | 0.64 (0.54-0.76) | <0.001 | 0.63 (0.54-0.74) | <0.001 | 0.70 (0.57-0.88) | 0.002 |
| Alb (g/L) | 1.11 (1.08-1.15) | <0.001 | 1.03 (0.99-1.08) | 0.129 | 1.15 (1.09-1.22) | <0.001 | 1.03 (0.95-1.11) | 0.435 |
| eGFR (mL/min/1.73m^2^) | 1.01 (1.00-1.02) | <0.001 | 1.00 (0.99-1.01) | 0.801 | 1.01 (1.00-1.02) | 0.004 | 1.00 (0.99-1.01) | 0.879 |
| M1 | 0.67 (0.34-1.32) | 0.252 |  |  | 0.90 (0.39-2.05) | 0.796 |  |  |
| E1 | 0.91 (0.65-1.28) | 0.606 |  |  | 0.91 (0.60-1.37) | 0.656 |  |  |
| S1 | 0.96 (0.68-1.35) | 0.807 |  |  | 0.99 (0.66-1.50) | 0.978 |  |  |
| T1/2 | 0.55 (0.38-0.80) | 0.002 | 0.75 (0.51-1.10) | 0.145 | 0.61 (0.39-0.94) | 0.027 | 0.72 (0.45-1.17) | 0.182 |
| C1/2 | 1.37 (0.99-1.90) | 0.060 | 1.31 (0.94-1.84) | 0.112 | 1.23 (0.82-1.84) | 0.315 | 1.27 (0.84-1.94) | 0.261 |
| Hypertension | 0.91 (0.62-1.33) | 0.610 |  |  | 0.90 (0.57-1.43) | 0.659 |  |  |
| Diabetes | 0.83 (0.37-1.89) | 0.659 |  |  | 0.58(0.21-1.60) | 0.295 |  |  |

Abbreviations: Alb: serum albumin; BMI: body mass index; C: crescent; DBP: diastolic blood pressure; E: endocapillary hypercellularity; eGFR: estimated glomerular filtration rate; M: mesangial hypercellularity; S: segmental glomerulosclerosis; T: interstitial fibrosis and tubular atrophy.
